# Diagnosis and cardiac transplantation of a Carney syndrome-induced cardiac myxoma combined with dilated cardiomyopathy: a case report

**DOI:** 10.1186/s12872-024-03946-4

**Published:** 2024-06-17

**Authors:** Cheng Cheng, Yang Song, Haidong Yan, Daojun Bao, Xiaodi Zhang, Yi Zhao, Daxing Liu, Dengshen Zhang

**Affiliations:** 1 Department of Cardiovascular Surgery, Affiliated Hospital of Zunyi Medical University, Zunyi, Guizhou, 563000 China; 2 Department of Ultrasound, Affiliated Hospital of Zunyi Medical University, Zunyi, Guizhou, 563000 China

**Keywords:** Carney syndrome, *PRKAR1A* gene mutation, Cardiac myxomas, Dilated cardiomyopathy, Heart transplantation, Heart failure

## Abstract

**Background:**

Carney syndrome is an uncommon autosomal disorder closely linked to mutations in the PRKAR1A gene. Skin lesions are the most pronounced feature of Carney syndrome, affecting over 80% of individuals with this condition. This syndrome is characterized by a triad of myxomas, skin pigmentation, and endocrine hyperfunction, featuring multiple endocrine neoplasms with skin and cardiac involvement.

Dilated cardiomyopathy, a primary cardiomyopathy, is defined as the dilation and impaired systolic function of the left or both ventricles. Its clinical presentation varies from being asymptomatic to heart failure or sudden cardiac death, making it a leading global cause of heart failure. Currently, Dilated cardiomyopathy has an estimated prevalence of 1/2500–1/250 individuals, predominantly affecting those aged 30–40 years, with a male-to-female ratio of 3:1. This case report describes a heart failure patient with cardiac myxoma caused by Carney syndrome combined with dilated cardiomyopathy. The patient was successfully treated for heart failure by heart transplantation.

**Case presentation:**

Herein, we report a case of heart failure due to Carney syndrome that resulted in cardiac myxoma combined with dilated cardiomyopathy. A 35-year-old male was admitted to the hospital three years ago because of sudden chest tightness and shortness of breath. Echocardiography indicated myxoma, and a combination of genetic screening and physical examination confirmed Carney syndrome with cardiac myxoma. Following symptomatic management, he was discharged. Surgical interventions were not considered at the time. However, the patient’s chest tightness and shortness of breath symptoms worsened, and he returned to the hospital. A New York Heart Association grade IV heart function was confirmed, and echocardiography indicated the presence of dilated cardiomyopathy accompanied by cardiac myxoma. Ultimately, the patient’s heart failure was successfully treated with heart transplantation.

**Conclusions:**

Cardiac myxoma caused by Carney syndrome combined with heart failure caused by dilated cardiomyopathy can be resolved by heart transplantation.

**Supplementary Information:**

The online version contains supplementary material available at 10.1186/s12872-024-03946-4.

## Background

Carney syndrome (CNC) is a rare autosomal genetic disorder with mutations in the *PRKAR1A* gene implicated in its pathogenesis [1]. CNC is characterized by cardiac myxomas, skin pigmentation, and endocrine system overactivity. The key features include multiple endocrine tumors as well as skin and heart involvement. Typically, patients with CNC develop skin pigmentation manifesting as brown or black spots ranging from 2 to 10 mm in diameter, often found on the lips, eyelids, ears, and genitalia [2].

Dilated cardiomyopathy (DCM) is a primary myocardial disease characterized by dilation and impaired systolic function of the left or bilateral ventricles, although its cause remains largely unknown [3]. Clinical presentation varies widely, ranging from asymptomatic to heart failure or sudden cardiac death [4]. DCM has an estimated prevalence of 1/2500–1/250 individuals [5], predominantly affecting individuals aged 30–40, with a male-to-female ratio of 3:1 [6]. DCM is one of the leading causes of heart failure worldwide. Heart transplantation is the preferred treatment option for patients with end-stage heart failure due to DCM [7].

## Case presentation

**Fig. 1:**
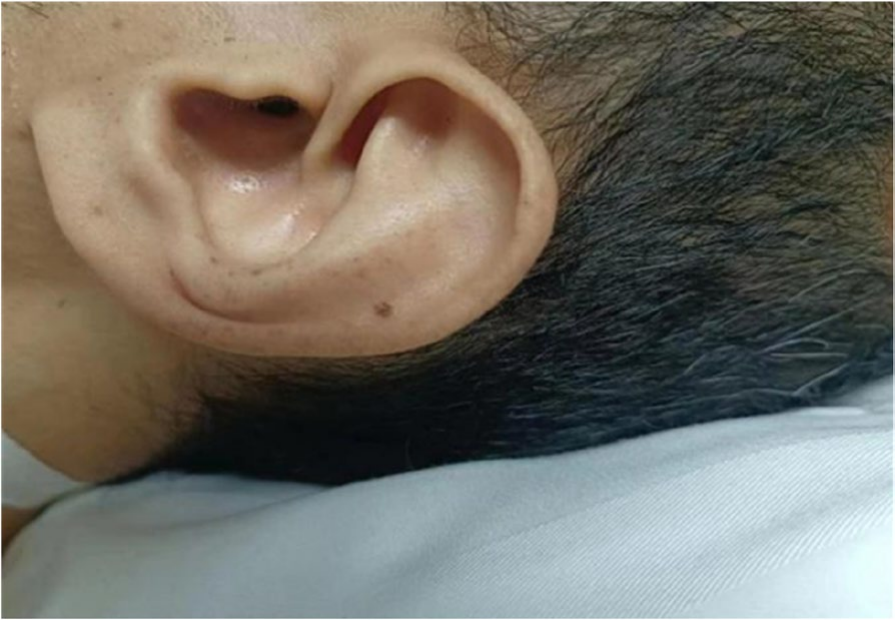
Examination of the patient's face revealed hyperpigmentation of the skin in the ear, with multiple small brown and black spots

**Fig. 2:**
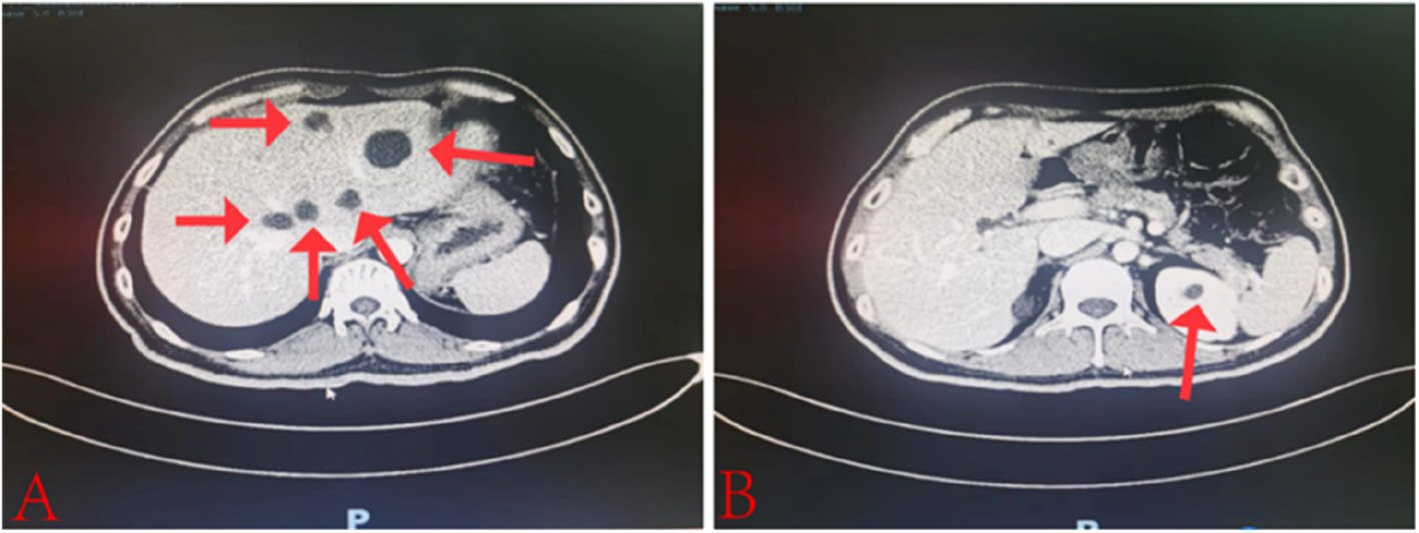
Panel **A** shows a liver cyst (arrow), while Panel **B** illustrates a cyst in the left kidney (arrow)

**Fig. 3:**
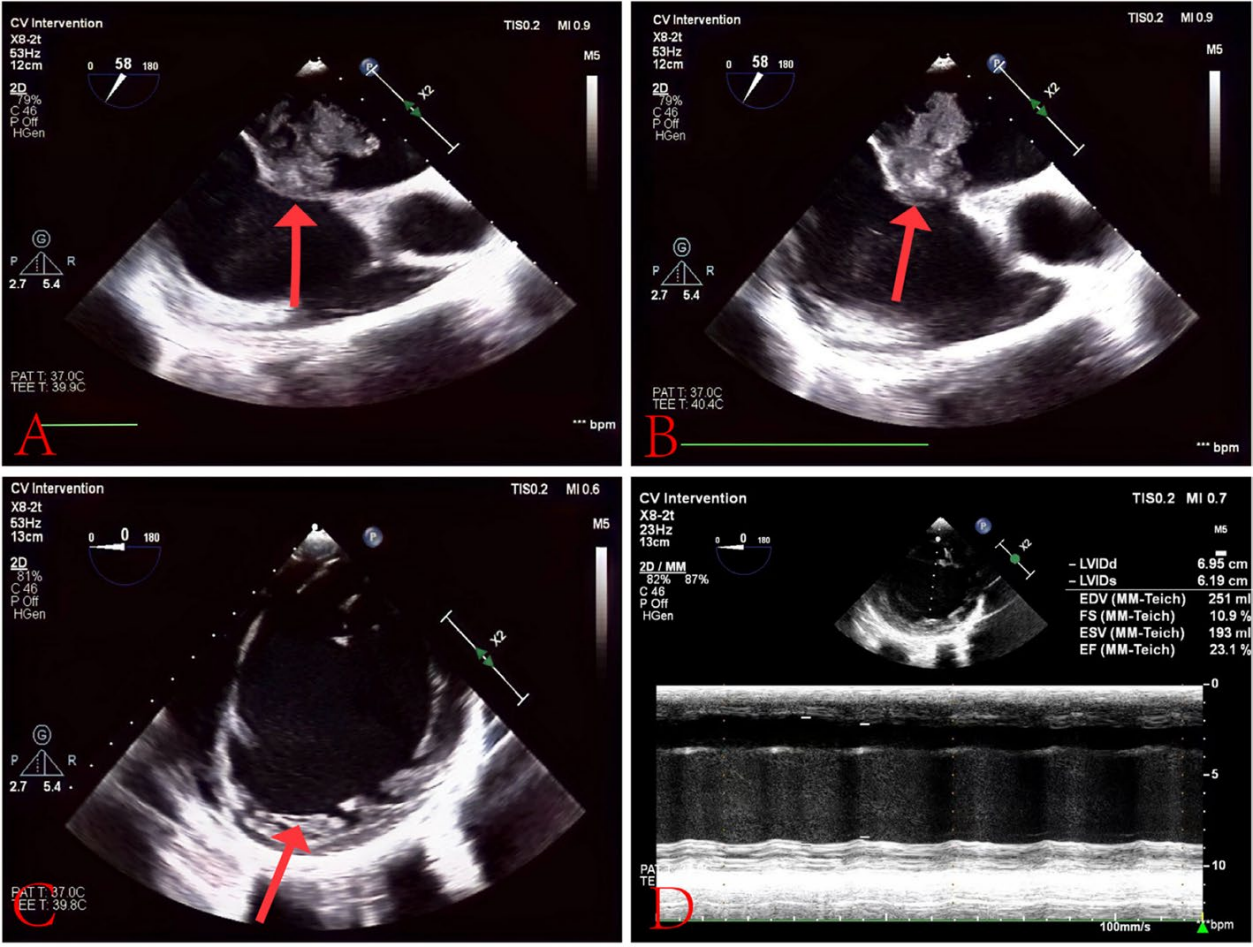
Panels **A** and **B** highlight a myxoma in the left atrium (indicated by arrows). Panel **C** shows an enlarged heart (arrow). Panel **D** provides measurements: left ventricular end-diastolic diameter at 6.95 cm; left ventricular end-systolic diameter at 6.19 cm; end-diastolic volume at 251 ml; fractional shortening at 10.9%; end-systolic volume at 193 ml; and ejection fraction at 23.1%. Conclusion: left ventricular echo mass was considered as myxoma. Total heart enlargement, reduced left ventricular wall motion, considered dilated cardiomyopathy

**Fig. 4:**
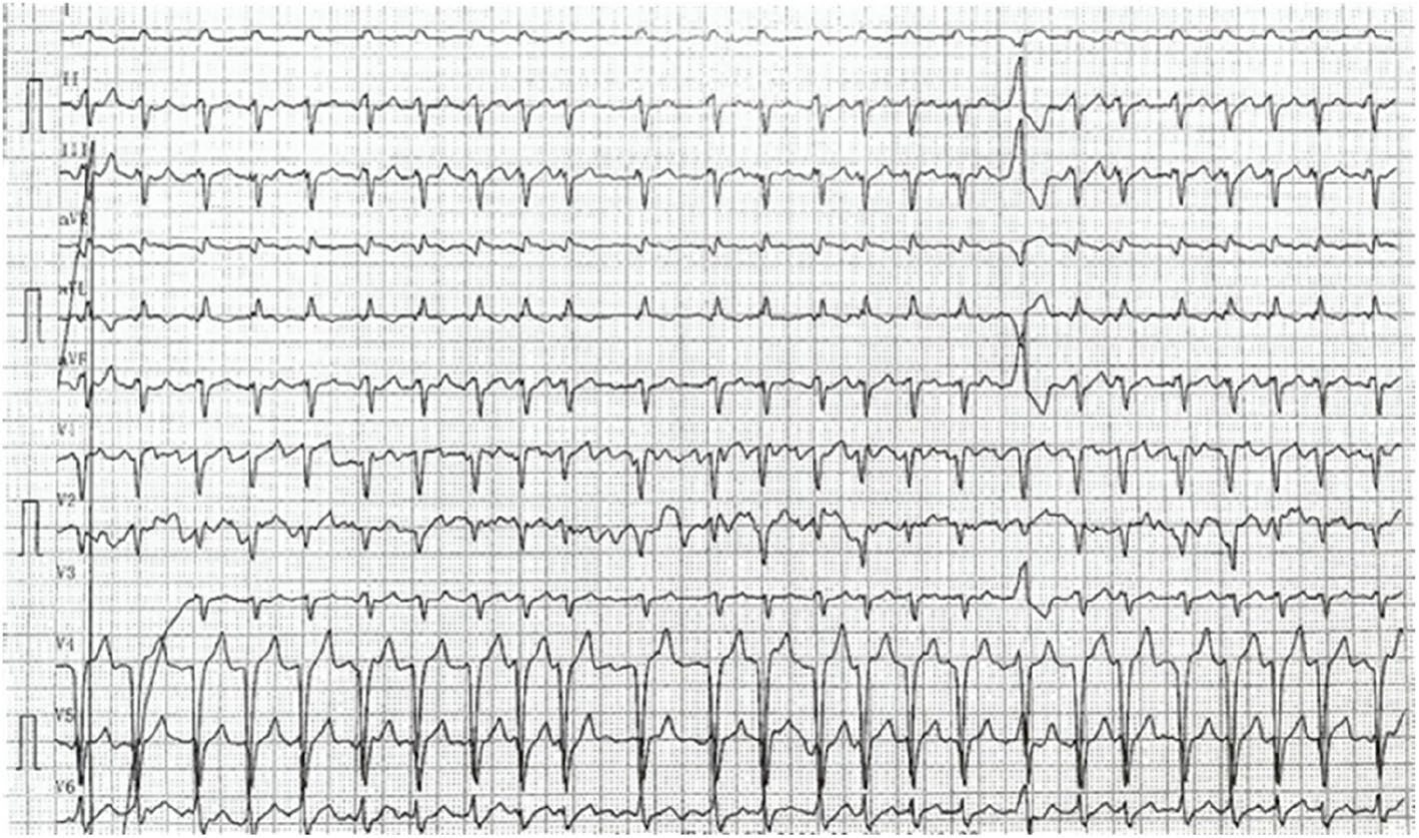
(1) The ectrocardiogram revealed atrial fibrillation (average ventricular rate of 150 beats/min). (2) Abnormal Q waves in leads V1-V3. (3) Left anterior branch block. (4) Ventricular premature beat

A 38-year-old male presented to the hospital with chest tightness and shortness of breath. Three years prior, he had experienced similar symptoms post-activity and received treatment at our hospital. Outpatient echocardiography indicated a left heart echomass suggestive of a myxoma, which led to his admission for further evaluation. Physical examination revealed pigmentation of the patient’s ears characterized by multiple small brown and black spots (Fig. 1). Abdominal computed tomography (CT) showed multiple livers and small cysts in the left kidney (Fig. 2). Genetic testing identified mutations in the TTN and PRKAR1A genes (Table 1). The diagnosis of CNC was confirmed through clinical examination, imaging, and genetic testing (Table 2) [8]. Following symptomatic treatment, the patient’s condition improved; however, he refused surgical intervention. On September 20, 2023, the patient presented to our hospital with exacerbated chest tightness and dyspnea. He reported difficulty lying supine and needing to sit upright to breathe. Physical examination revealed jugular vein distension, leftward and downward displacement of the heart boundary, irregular heart rhythm on auscultation, and a mitral valve murmur of intensity 2/6–3/6 in the fourth intercostal space along the left sternal margin. Wet rales were audible in both middle and lower lung fields. Palpation revealed a firm liver extending three fingers below the xiphoid process and two fingers below the rib cage, along with mild pitting edema in both lower limbs. Echocardiographic images indicated global heart enlargement, dilation of the aortic sinus and pulmonary artery, small-to-moderate mitral valve regurgitation, and an irregular echoic mass measuring 54 mm ×43 mm in the left chamber attached to the atrial septum. The left ventricular (LV) ejection fraction (EF) was 23.1%, with fractional shortening (FS) of 10.9% (Fig. 3). Electrocardiography demonstrated atrial fibrillation (average ventricular rate, 150 beats/min) and abnormal Q waves in leads V1-V3 (Fig. 4). Based on the patient’s history, the diagnosis included DCM and CNC with cardiac myxoma. Given the presence of end-stage heart failure and concurrent cardiac myxoma, the patient was hospitalized, and heart transplantation was considered a viable therapeutic option to address both conditions simultaneously. A suitable donor heart became available for immediate transplantation on October 1, 2024.

**Table 1:** This image represents the patient’s monogenic genetic disease test report issued by Beijing Nuohexin Kang Gene Technology Co., Ltd

Variant gene	Nucleotide variant	Amino acid variation	Transcript exon	Variable state	Type of mutation	ACMG entry	Pathogenicity	Associated diseases (Patterns of inheritance)
TTN	C.89,201–2 A > G	Uncertain	NM_133378exon97	Hybridization	Shear zone variation	PM2 PVS1	Might cure the disease.	Arrhythmogenic right ventricular cardiomyopathy (unknown) Hypertrophic cardiomyopathy type 9 Dilated cardiomyopathy type 1G (unknown)
PRKAR1A	C.637delG	P.Ala213profs*9	NM_002734exon7	Hybridization	Shift Code Missing Variant	PM2 PVS1	Might cure the disease.	Acromegaly type 1 with or without hormonal resistance (AD) Primary pigmented nodular adrenocortical disease type 1, PPNAD1 (AD) Intracardiac Mucinous Tumor (AD) Carney syndrome type 1 (AD)

**Table 2:** This figure displays the diagnostic chart for Carney Complex (CNC) disease

Diagnostic basis	Characteristic
Main features	1. Skin pigmentation is punctate and found in various areas, such as the lips, conjunctiva, inner and outer canthus, vagina, and penile mucosa.
	2.Myxoma (skin, mucosa) or cardiac myxoma
	3.A breast myxoma can be confirmed through a fat suppression MRI scan.
	4. An abnormal increase in urinary glucocorticoids in response to dexamethasone in primary pigmented nodular adrenal cortical disease or the Liddle test.
	5. Acromegaly is a medical condition resulting from adenomas that persist in secreting growth hormones.
	6. Male individuals may present with testicular large cell calcified Sertoli cell tumors, confirmed either by a testicular ultrasound examination revealing characteristic calcification or through pathology results. Female individuals may present with either ovarian cancer or ovarian cysts.
	7. Ultrasound examination of patients with thyroid cancer or prior to puberty indicates multiple hypoechoic nodules within the thyroid gland.
	8.Sandy black schwannoma
	9.Multiple blue nevus or epithelioid blue nevus
	10. Multiple Ductal Adenomas of the Breast
	11.Osteochondromyxoma
Supplementary features	1.First-degree relatives of patients with CNC.
	2.Mutations in PRKACA, including single base substitutions and copy number variations, as well as activation mutations in PRKACB, have been observed.
	3.Inactivation mutation of PRKAR1A gene
Diagnostic criteria	1.Meet two or more major characteristics
	2.Conform to one or more supplementary features, and conform to one or one main feature at the same time

### Surgical procedure

**Fig. 5:**
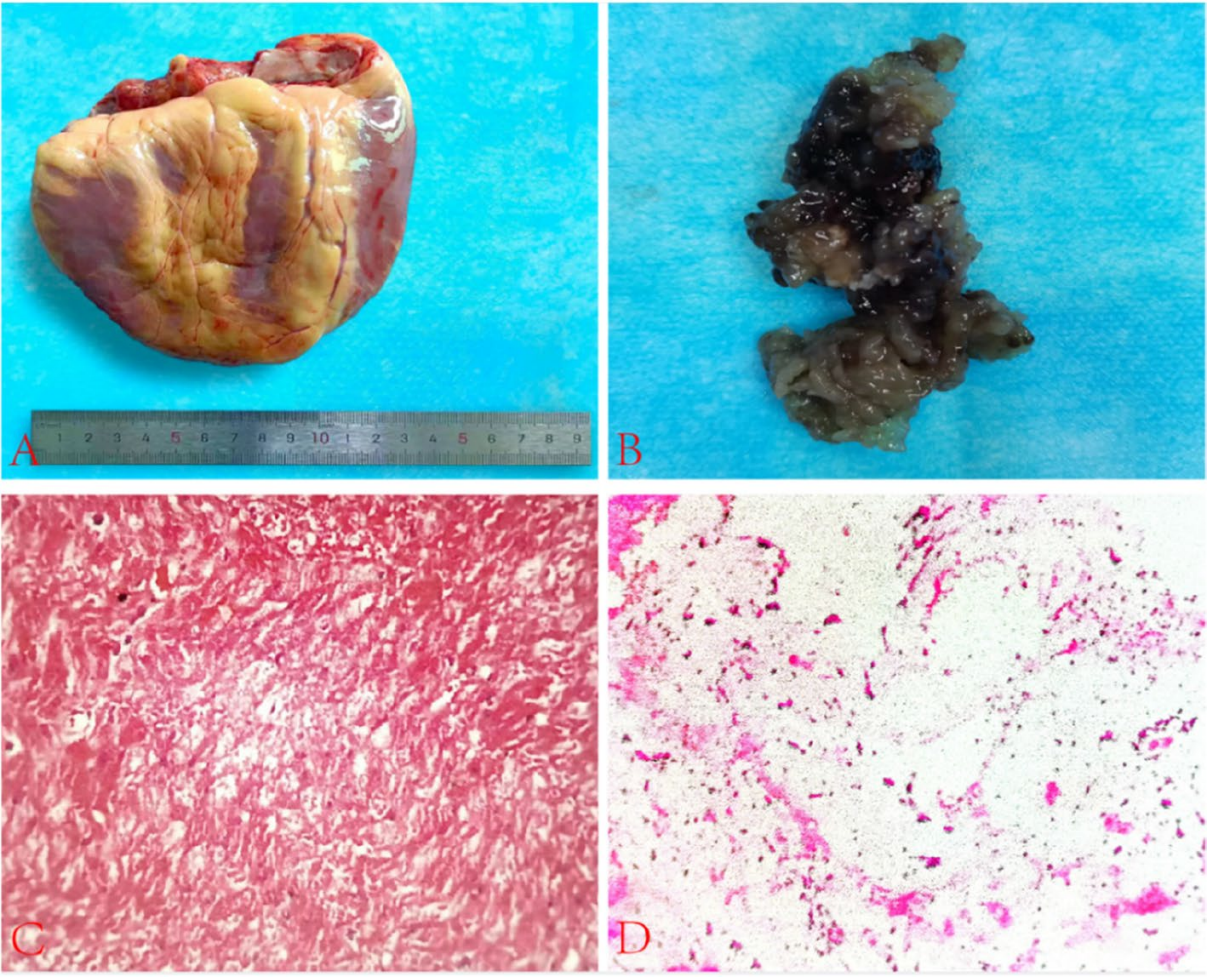
Panel **A** The patient's heart was removed after surgery, with a width of approximately 13 cm; Panel **B** The myxoma was grayish white after surgical resection and cleaning with normal saline. Panel **C** displays heterogeneous myocardial hypertrophy, deep staining of cell nuclei, irregular nuclear shapes, atrophy of some myocardial cells, vacuolar degeneration in parts of the myocardium, and interstitial fibrosis, consistent with the pathological changes of dilated cardiomyopathy. Panel **D**: Gray jelly-like tissue shows a left atrial myxoma

The skin and subcutaneous tissues were carefully incised layer-by-layer through a median sternotomy. The sternum was sawed longitudinally open, and bleeding was controlled using electrocoagulation and bone wax. Extracardiac exploration uncovered global heart enlargement, most prominent in the LV (Figure 5A). The heart showed diminished contractile strength. The aorta and the main pulmonary artery (PA) were dissected from the supravalvular region. Some tissues were preserved for posterior suturing, whereas most diseased right atrium, left atrium(LA), right ventricle, and LV were excised. Resection revealed a greyish-white mucoid mass (Figure 5B). The donor and residual recipient LA tissues were sutured using double continuous 3/0 Prolene threads. The anastomosis was meticulously inspected multiple times, and no significant bleeding was observed. Similarly, end-to-end anastomosis of the donor ascending aorta and recipient PA was performed using continuous 5/0 Prolene sutures, and careful inspection revealed no bleeding.

Furthermore, the donor’s LA and recipient’s PA were securely closed using double continuous 5/0 Prolene sutures. The inferior vena cava tissues of both the donor and recipient were similarly sutured with 5/0 Prolene sutures, and several inspections were performed to confirm no significant bleeding was present. The left side of the heart was then deflated, and as rewarming commenced, oxygenation was restored, the ascending aorta was unclamped, and the heart spontaneously returned to sinus rhythm. Continuous suturing with 5/0 Prolene was applied to both the donor and recipient’s superior vena cava and diligently inspected to ensure the absence of significant bleeding. After the successful discontinuation of assisted circulation, the venous cavity was decannulated. Tissue samples from the patient’s left heart and gray matter were collected for histopathological examination, and the diagnosis of DCM (Fig. 5C) and cardiac myxoma were confirmed (Figure 5D).

### Postoperative management

**Fig. 6:**
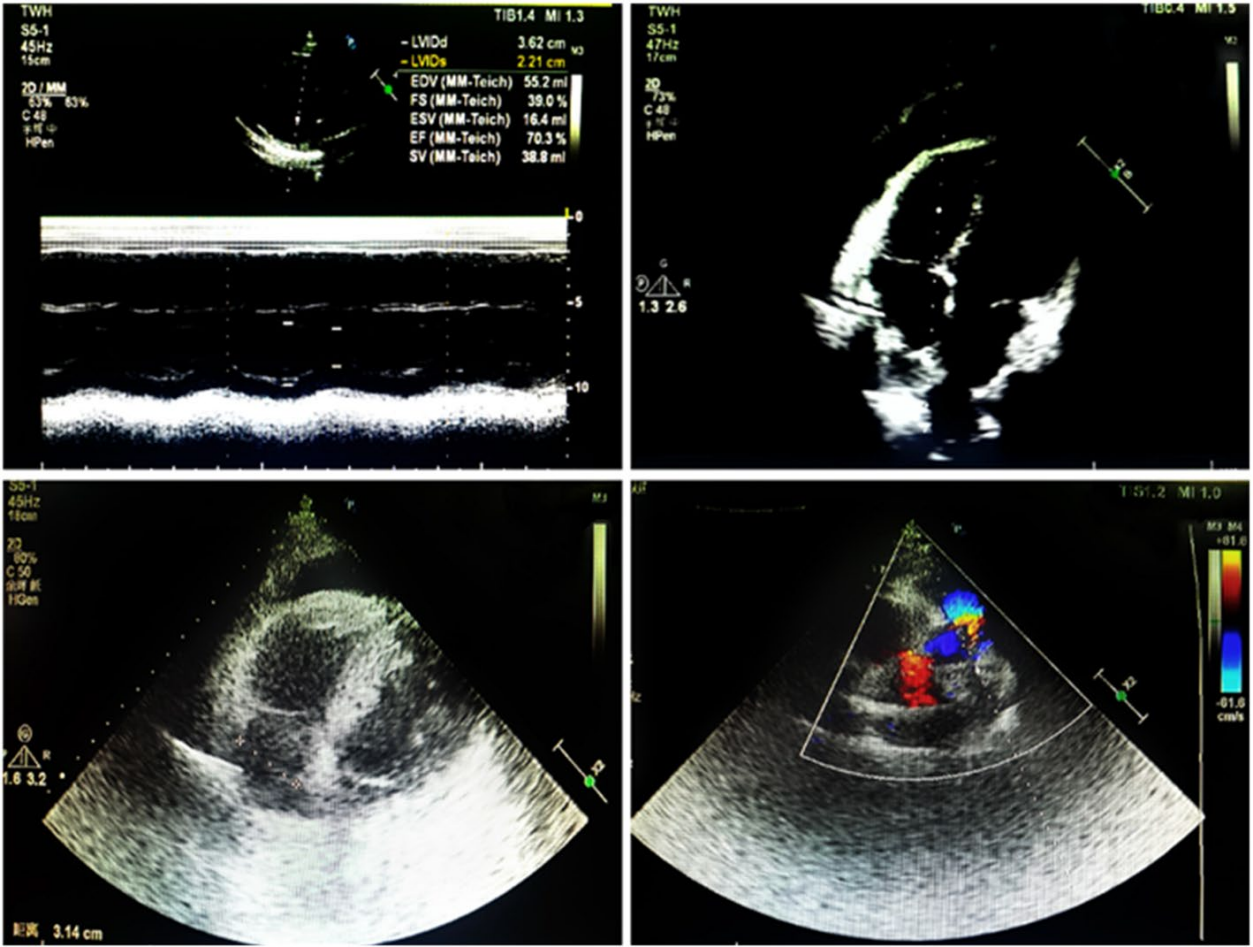
After heart transplantation: normal range of intra-atrial diameter. Coin thickness and echo are normal. No abnormalities were found in the valve mentality mechanism

On the first day after heart transplantation, the patient produced 1200 ml of urine. Laboratory tests revealed a hypersensitive troponin T level of 796.70ng/L and an NT-proBNP level of 10798pg/ml. The complete blood count showed white blood cells at 17.15 × 10^9^/L, with no significant abnormalities in other test results. The echocardiograph displayed an LVEF of 65%, FS of 35%, normal ventricular wall thickness and echogenicity, and no discernible abnormalities in valve morphology and structure. After heart transplantation, Methylprednisolone Sodium Succinate (0.25 g)intravenous hormone therapy was administered to enhance immunity, and Cefoperazone and Sulbactam Sodium (2 g) intravenous anti-infection treatment was provided. The patient was given a nutrient solution and liver and tiopronin on the first day post-surgery. On postoperative day three, Methylprednisolone Sodium Succinate was replaced with oral Prednisone Acetate (25 mg). Mycophenolate Mofetil capsules (0.5 g) were administered orally to minimize heart rejection, and (50 mg) of Carpofungin Acetate was administered intravenously to prevent fungal infections. The patient’s urine output was 2000 ml, with hypersensitive troponin T levels of 390ng/L, NT-proBNP levels of 7877pg/ml, and a leukocyte count of 12.15 × 10^9^/L. On the 7th day post-surgery, tacrolimus capsules were introduced at an oral dose of (1 mg) to minimize the patient’s rejection of the donor heart, with careful monitoring of blood concentrations. Subsequently, the oral dosage of Prednisone Acetate was gradually decreased to (10 mg) while adjusting the tacrolimus blood concentration to 10.90ng/ml. The patient’s recovery improved. On October 20, 2023, follow-up echocardiography (Fig. 6) indicated no abnormalities, with troponin levels of 85 ng/L, NT-proBNP of 210pg/ml, and all other test results within normal ranges. The patient exhibited excellent postoperative recovery and was discharged. Regular follow-up visits to our department after discharge showed that the patient remains in good condition.

## Discussion

CNC is a rare autosomal genetic disorder. Statistical analysis of the largest global dataset of CNC patients indicated that women represented 63% of the cases, while men represented 37%. Over 70% of CNC are linked to mutations in PRKAR1A (OMIM 188,830), which encodes the type III regulatory subunit of protein kinase A (PKA, cAMP-dependent protein kinase), also known as CNC1 [9]. Skin lesions are the most pronounced features of CNC, affecting over 80% of individuals [10]. Skin pigmentation typically manifests as brown or black spots ranging from 2 to 10 mm in diameter, frequently observed on the lips, eyelids, ears, and genitals. Beyond skin manifestations, Cardiac myxoma is a significant symptom of CNC, typically manifesting by the age of 20, though it can also occur in infancy [2, 10]. Tumors may develop anywhere in the heart, with the LA being the most common site, and are often characterized by multiplicity and recurrence. The clinical symptoms vary depending on the presence of intracardiac blood flow obstruction, thromboembolism, or chronic heart failure. Complete obstruction of the valve by a tumor can result in sudden death. Additionally, CNC is associated with multiple endocrine tumors and cysts, including adrenal cortical tumors, thyroid neoplasms, liver cysts, and kidney cysts [1].

DCM is characterized by dilation and impaired contraction of the left or both ventricles [11]. It typically manifests between the ages of 30 and 40, with a higher prevalence in men than in women. DCM’s multifactorial etiology involves viral infections, genetic factors, autoimmunity, and cellular immune responses [6]. Advances in molecular genetics have led to a refined classification of cardiomyopathies as primary or secondary based on their genetic underpinnings. Primary DCM can be categorized as familial dilated cardiomyopathy (FDCM) or acquired DCM. Approximately 60% of FDCM cases are genetically linked to one of the 60 DCM-associated genes [12]. Molecular genetic studies have identified “causal” mutations in over 60 genes related to DCM, including significant findings such as truncating mutations in the TTN gene in familial or severe cases requiring transplantation [13] and TTN truncation in about 13% of non-familial DCM cases [14]. Recent research on DCM pathogenesis underscores that the TTN gene is the primary diagnostic marker for FDCM, accounting for approximately 30–35% of cases [15].

A 37-year-old male with TTN and PRKAR1A variants was definitively diagnosed with CNC-induced Cardiac myxoma and DCM. CNC has no specific treatment, and the management primarily involves symptomatic care. However, patients with cardiac myxoma require prompt surgical removal as surgery is the primary treatment method [16]. DCM often progresses to end-stage heart failure, for which heart transplantation is the preferred treatment [17]. Given the patient’s progression to terminal heart failure, compounded by CNC-related Cardiac myxoma, heart transplantation was considered to address the cardiac myxoma, DCM, and overall heart failure simultaneously. The median survival after heart transplantation in adults stands at 10.7 years [18]. Our team performed a heart transplantation to remove diseased tissue and transplanted a new donor heart. After the operation, the patient took Mycophenolate Mofetil capsules and tacrolimus capsules orally to reduce immune rejection of the donor’s heart. The patient subsequently recovered well. This case is unique because the CNC caused cardiac myxoma combined with DCM. There have been no reports of these two diseases occurring simultaneously in a single patient. Although heart transplantation resolved the patient’s heart failure symptoms, the patient still had CNC and carried a mutated gene. It is not yet known whether CNC affects the outcome of heart transplantation or whether the transplanted donor heart develops myxoma again. We will continue to track patient outcomes to elucidate an answer.

## Summary

CNC is an uncommon autosomal genetic disorder characterized by cardiac myxomas, skin pigmentation, and endocrine overactivity, involving multiple endocrine tumors as well as the skin and heart. DCM is a primary cardiac disease characterized by impaired dilation and systolic function of the left or double ventricles. Clinical manifestations range from asymptomatic to heart failure or sudden cardiac death, and it is one of the most common causes of heart failure worldwide. Here, the patient had CNC-induced Cardiac myxoma in conjunction with DCM. The diagnosis of DCM is generally straightforward, but the presence of skin pigmentation, multiple tumors, or cardiac myxoma warrants careful consideration of CNC. There is no specific cure for CNC, and treatment primarily focuses on symptom management. Early surgical removal of cardiac myxomas is crucial for affected patients. Heart transplantation remains the treatment of choice for patients with DCM who progress to end-stage heart failure. In this case, the patient’s heart failure, induced by both DCM and a CNC-related Cardiac myxoma, was successfully treated with a heart transplant that involved removing the diseased tissue and replacing it with a healthy donor heart. Eventually, the patient’s symptoms of heart failure resolved.

### Supplementary Information

Supplementary Material 1.

## Data Availability

All relevant data supporting the conclusions of this article are included within the article.

## Abbreviations

DCM: Dilated cardiomyopathy
CNC: Carney syndrome
LV: Left ventricular
LA: Left atrium
CT: Computed tomography
PA: Pulmonary artery
EF: Ejection fraction
FS: Fractional shortening
FDCM: Familial dilated cardiomyopathy
