# Supplementary material for: Diagnosis and cardiac transplantation of a Carney syndrome-induced cardiac myxoma combined with dilated cardiomyopathy: a case report
Source: BMC Cardiovasc Disord. 2024 Jun 17;24:307. doi: 10.1186/s12872-024-03946-4 (PMC11181650; doi:10.1186/s12872-024-03946-4)
Supplement: Supplementary file 1 — Supplementary Material 1. [file 12872_2024_3946_MOESM1_ESM.docx]

Supplementary images on dilated cardiomyopathy and myxoma
